# Immunogenic Cell Death and Elimination of Immunosuppressive Cells: A Double-Edged Sword of Chemotherapy

**DOI:** 10.3390/cancers12092637

**Published:** 2020-09-16

**Authors:** Jean-David Fumet, Emeric Limagne, Marion Thibaudin, Francois Ghiringhelli

**Affiliations:** 1 Department of Medical Oncology, Center GF Leclerc, 21000 Dijon, France; fghiringhelli@cgfl.fr; 2 Platform of Transfer in Cancer Biology, Center GF Leclerc, 21000 Dijon, France; elimagne@cgfl.fr (E.L.); mthibaudin@cgfl.fr (M.T.); 3 University of Burgundy Franche Compte, 21000 Dijon, France; 4 UMR INSERM 1231 “Lipides Nutrition Cancer”, 21000 Dijon, France

**Keywords:** cancer, chemotherapy, immunotherapy, immunogenic cell death, immunosuppression

## Abstract

**Simple Summary:**

The aim of this review is to detailed immunological effects of chemotherapies focusing on 2 main effects: immunogenic cell death and depletion of suppressive cells. It provides a strong rational for combination of chemotherapy and immunotherapy.

**Abstract:**

Chemotherapy is initially used to kill proliferative cells. In the current area of emerging immunotherapy, chemotherapies have shown their ability to modulate the tumor micro environment and immune response. We focus here on two main effects: first, immunogenic cell death, defined as a form of regulated cell death (RCD) that is sufficient to activate an adaptive immune response in immunocompetent hosts; and second, the depletion of suppressive cells, known to play a major role in immune escape and resistance to immunotherapy. In this review, we present a review of different classically used chemotherapies focusing on this double effect on immunity. These immunological effects of chemotherapy could be exploited to promote efficacy of immunotherapy. Broadening our understanding will make it possible to provide rationales for the combination of chemoimmunotherapy in early clinical trials.

## 1. Background

Historically, carcinogenesis and cell death are two biologic processes defined by a specific sequence of molecular events. First, cancer arising from the carcinogenesis process was defined as a cell autonomous disease with an imbalance between proto-oncogene activation and/or inactivation of oncosuppressor genes, leading to uncontrolled cell proliferation and resistance to cell death. In this way, cytotoxic chemotherapy was developed with the major aim of killing proliferative cells. However, this strategy only aims to disrupt the cell death/proliferation balance and does not take into account physiological strategies for defense against carcinogenesis. This view of therapeutics in oncology has been revolutionized by new discoveries related to the antitumor immune response and the mechanisms associated with its inhibition by immune checkpoints. Indeed, carcinogenesis is no longer defined solely as genetic disturbances of the malignant cell that give it enhanced proliferation properties, but rather, its definition now also includes an alteration of the immune system that becomes permissive to tumor proliferation. Thus, the last decade has been characterized by the emergence of immunotherapy, with the development of a new strategy to fight cancer cells. The aim is not to destroy proliferating cells, but to improve the adaptive immune response to recognize and eliminate cancer cells. The major discovery pertains to immune checkpoints, which consist of membranous molecules physiologically expressed during T-cell activation and in inflammatory conditions. Immune checkpoint inhibitors drastically improve immune response, leading to improvement of antitumor immune response. Since the early 2010s, many immunotherapies based on the inhibition of immune-response control points have been developed and shown to be therapeutically superior to conventional chemotherapy. This is notably the case of antibodies targeting the CTLA4 or the PD-1/PD-L1 pathways, which have revolutionized the treatment of melanoma or lung cancer. For example, use of anti-PD-1 antibodies as monotherapy in metastatic non-small cell lung cancer (NSCLC), in second line or more, provides 20–30% lasting control of the disease [1]. Despite this revolutionary efficacy, many patients present an intrinsic resistance to immunotherapy, which may be explained by many biologic phenomena. Most of these tumors present an absence of lymphocyte infiltration (NK, CD8, Th1). It is now clearly established that the presence of this cytotoxic response is a prerequisite for the effectiveness of immunotherapy [2,3]. Biologic phenomena related to this lack of immune response include: (i) loss of antigenicity in tumor cells—linked to a restricted neoantigen level, the disappearance of antigenic presentation systems, or the inability of cells to respond to interferons, powerful inducers of antigenic presentation; (ii) absence or inhibition of danger signals linked to inflammatory and immune response in the tumor microenvironment; (iii) enrichment of regulatory immune cells blocking infiltration and cytotoxic functions of antitumor lymphocytes [4]. To modulate these phenomena, a combination of immunotherapy with chemotherapy has been proposed and clinically tested. In NSCLC, a significant survival benefit was shown in a Phase III study comparing the combination of pembrolizumab (anti-PD-1) and platinum/pemetrexed chemotherapy versus chemotherapy alone. The observed therapeutic effect was independent of the expression of PD-L1 by tumor cells [5]. The biologic and immunological rationale to explain the efficacy of this combination is based on the ability of chemotherapy to restore an immune response through several complementary mechanisms in non-infiltrated tumors. Indeed, there is an increasing body of evidence showing that chemotherapies can cause so-called “immunogenic” cancer cell death (ICD) [6], which can stimulate host antitumor immunity. In addition to the ICD process, chemotherapies could also affect immune response via their ability to eliminate certain cells, especially immunosuppressive cells, in the cancer microenvironment, thus leading to an improvement in antitumor immune response. These immunological properties enable chemotherapy to transform a non-inflammatory tumor, known as “cold”, into a tumor enriched with cytotoxic cells, known as “hot” [7]. This change in phenotype makes it possible to sensitize the tumor to checkpoint blockade, which requires a pre-existing immune response. Currently, some clinically approved therapies and clinical trials are using associations of chemotherapies and immunotherapies. In this context, improved knowledge of chemotherapy immune properties is indispensable to inform the development of effective combinations. Here, we propose an analysis of this double-edged sword of chemotherapy, with a special focus on ICD induction and elimination of immunosuppressive cells in order to design future combination strategies.

## 2. Chemotherapy Induced ICD

**Figure 1:**
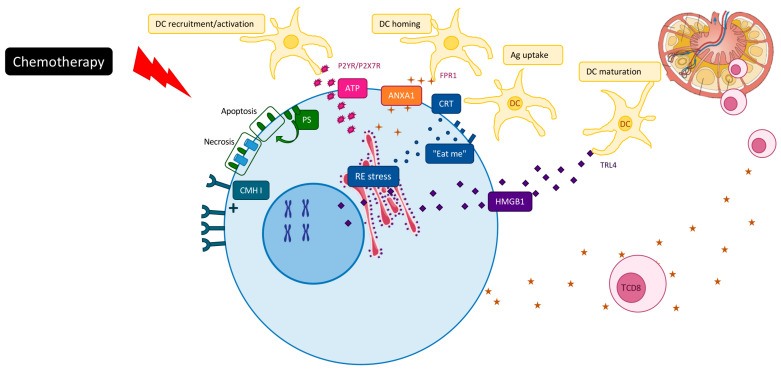
Mechanisms of chemotherapy-driven “immunogenic” cancer cell death (ICD).

In accordance with the recommendations of the nomenclature committee on cell death 2018, ICD has been defined as a form of regulated cell death (RCD) that is sufficient to activate an adaptive immune response in immunocompetent hosts [6]. ICD can be initiated by various stimuli, including viral infection, some chemotherapeutics or radiation therapy or cell death triggered by immune cells [8,9,10]. These agents are able to generate several danger signals, called danger-associated molecular patterns (DAMPs), which will be recognized by pattern recognition receptors (PRRs) expressed by immune cells, such as dendritic cells. To date, five DAMPs have been mechanistically linked to the perception of RCD as immunogenic: (1) calreticulin (CALR) [11,12], (2) ATP [13], (3) high-mobility group box 1 (HMGB1) [14,15], (4) Type I IFN [16] and (5) annexin A1 (ANXA1) [17] (Figure 1).

First of all, early translocation of endoplasmic calreticulin to the tumor cell surface generates an “eat-me” signal for DC phagocytosis and tumor antigen uptake [11]. Then, secretion of ATP stimulates macrophage and dendritic cell recruitment and their maturation [18], induces NK cell proliferation and stimulates IFNγ production via production of IL1β [19]. Post-apoptotic release of the nuclear chromatin binding protein HMGB1 activates Toll-like receptor 4 (TLR4) and mediates nucleic acid activation of TLRs 3, 7 and 9 [20]. It favors optimal cross presentation of the MHC Class I peptide complex to CD8 T cells. Annexin-1 binds to formyl peptide receptor 1 (FRP1) on dendritic cells, which then promote stable interactions between dying cancer cells and presenting cells [17]. ICD drugs can also activate type-I interferon signaling pathways in tumor cells, which promote CXCL10 production and immune recruitment [16]. It has been noted that while DAMPs represent one of the main events of ICD, in certain circumstances the presence of DAMPs could promote tumor progression. Increase DAMPs could cause excessive inflammation and immune injury [21]. The main open question is the difference between DAMPs that drive tumor progression and those that block tumor growth [22].

Based on a classification related to their principal mechanism of action, we present below in further detail the impact of each type of chemotherapy on ICD (Table 1).

### 2.1. Alkylating Agents and Platinum Derivatives

These cytotoxic agents cause inter- or intra-strand DNA crosslinks that destabilize DNA during replication. Cyclophosphamide, an alkylating agent, induces antitumor immunity with ICD [48,49]. Platinum-based therapies exhibit a different capacity to trigger ICD [23]. The immunogenic properties of cisplatin are controversial and probably depend on the tumor type. Cisplatin was originally described as unable to elicit ICD because of its incapacity to promote the ER stress-dependent exposure of CALR on the cell surface and HMGB1-dependent TLR4 stimulation in colon or lung cancer models [50,51]. This incapacity can be corrected by the administration of agents that stimulate ER stress response, such as digoxin [52]. Moreover, in a clinical setting, cisplatin is frequently administered together with radiotherapy, which is known to promote ICD. Therefore, this combination could be used to enhance immune response via ICD [11]. Nevertheless, cisplatin can act on antigen presentation by inducing expression of Class I molecules and enzymes linked to tumor antigen processing. Moreover, in a context of triple negative breast cancer, cisplatin is associated with antitumor immune response and an ability to sensitize the tumor to PD-1 blockade [25]. Carboplatin showed a moderate ability to induce CALR exposure and HMGB1 release [27]. Interestingly, oxaliplatin is a strong ICD inducer, which may contribute to its clinical efficacy [53]. Moreover, it can stimulate immune response and promote the efficacy of immune checkpoint inhibitors [54,55]. Overall, alkylating agent are not equal on ICD effect. Oxaliplatin appears to be the best ICD inducer among platinum therapies.

### 2.2. Anti-Metabolites Inhibit the Synthesis of DNA and RNA

This large family is composed of agents that inhibit the synthesis of DNA and/or RNA. Gemcitabine is not considered as an immunogenic chemotherapeutic agent, but could potentiate immune response by stimulating the expression of MHC Class I by cancer cells [29] and it improves release of HMGB1 [32].

The ICD impact of 5-fluorouracil (5-FU), a key drug in gastrointestinal cancer, remains debated, with controversial results about calreticulin exposure [33,56] and HMGB1 release in some cancer models [35].

Trifluridine/tipiracil, also known as TAS-102, is a new orally active antimetabolite agent used to treat chemorefractory metastatic colorectal cancer. We have previously shown that in vitro, TAS-102 induced expression of all ICD markers in CT26 cells. Furthermore, the combination of TAS-102 and oxaliplatin was synergistic and enhanced ICD induction [36].

Pemetrexed, classically used in NSCLC adenocarcinoma, has also shown in vitro its ability to induce ICD, with significant release of HMGB1 [37], calreticulin exposure and interferon Type I [38].

Overall, with the exception of gemcitabine, antimetabolites appear to be interesting ICD inducers in many models.

### 2.3. Topoisomerase Inhibitors

Topoisomerase inhibitors impede the correct unwinding of DNA during replication and transcription. Mitoxantrone, doxorubicin and other anthracyclines and topoisomerase 2 inhibitors are strong ICD inducers, frequently used as a positive control for ICD analysis [39,41]. Bleomycin, which can be linked to this family, has exhibited a greater ability to trigger ICD [42] but was also associated with proliferation of regulatory T cells.

There are few data concerning topoisomerase 1 inhibitors. Irinotecan enhanced HMGB1 release in vitro [57] but its effect on other ICD determinants is unknown. Topotecan could not induce ICD, but stimulated tumor cells to produce IFN-β, which in turn operates as an autocrine factor to stimulate MHC Class I expression [58]. Among topoisomerase inhibitors, topoisomerase 2 inhibitors are widely described and are known to be strong ICD inducer. There is a lack of data concerning topoisomerase 1 inhibitors.

### 2.4. Microtubule Poisons

Paclitaxel and docetaxel are the main drugs in this family, which alters polymerization or depolymerization of tubulin.

Paclitaxel stimulates TLR4 [59], boosting T cell priming by DC [46]. A recent publication has shown that paclitaxel induced multiple molecular determinants of ICD via TLR4-independent and dependent pathways in ID8 mouse ovarian cancer cells [43]. These results observed in a murine model do not seem to be transposable to human cancers, because paclitaxel does not activate TLR4 in humans. Docetaxel appears to be less potent as an ICD inducer. It demonstrated an ability to enhance calreticulin exposure, but no release of ATP and HMGB1 [34]. Surprisingly, there are no data concerning the ICD impact of eribulin and vinorelbine. Overall, this family does not seem to be very effective on ICD induction. More data about eribulin and vinorelbine are needed.

## 3. Chemotherapy Eliminates Immunosuppressive Cells

Immunosuppressive cells, including myeloid derived suppressor cells (MDSC), regulatory T cells (Treg) and tumor associated macrophages (TAM), play a major role in antitumor immune response and are the main actors in immune escape and resistance to immunotherapy [60]. Interestingly, some chemotherapies have been shown to deplete immunosuppressive populations, thus restoring efficient antitumor immune response.

### 3.1. Depletion of MDSC

Gemcitabine and 5FU are the two most commonly known antimetabolite chemotherapies to deplete MDSC. This family effect is explained by lower expression by MDSC of thymidylate synthase and cytidine deaminase, the respective targets of 5FU and gemcitabine [61,62]. Our group has tested several cytotoxic chemotherapies, including cyclophosphamide, paclitaxel, raltitrexed, gemcitabine, doxorubicin, 5FU and oxaliplatin. Only 5FU and gemcitabine significantly decreased the number of MDSC in the tumor microenvironment. This elimination leads to increased interferon gamma production by CD8 cells, enhancing T-cell dependent antitumor response [61]. Despite controversial results [30,56], a growing body of data from different teams indicates that 5FU seems to impact on MDSC accumulation [31,63]. Interestingly, this effect is maintained with oxaliplatin [54], but is neutralized when 5FU is associated with irinotecan, which blocks MDSC death [31].

Gemcitabine inhibits and decreases MDSC number in the setting of 4T1 mammary carcinoma, resulting in T-cell expansion [44]. In another study in pancreatic cancer, gemcitabine was shown to decrease MDSC, especially granulocytic MDSC [47].

Docetaxel was shown to impair MDSC suppressive function by blocking STAT3 phosphorylation and by promoting MDSC differentiation into M1 macrophages [64]. Similarly, paclitaxel depletes MDSC [24,40] because of the presence of cytochrome P450 isoform in MDSC that converts paclitaxel into its active metabolite (6-a-hydroxypaclitaxel).

Doxorubicin eliminates and inactivates MDSC [28]. This preferential sensitivity of MDSC to doxorubicin could be explained by their high proliferation index.

Platinum therapies have various effect on MDSC. cisplatin [26] and oxaliplatin [65] have been shown to deplete MDSC. Conversely, carboplatin induced MDSC activation and enhanced exhaustion of CD8 T-cells [66].

### 3.2. Depletion of T-Regs

CD4^+^CD25^high^CD127^low^ T-regs represent a subpopulation of T-cells characterized by the expression of the master controller transcription factor FOXP3, essential for their development and function. Several chemotherapies have been found to exert an off-target effect on T-regs.

Cyclophosphamide, and especially a metronomic regimen, depletes T-regs, resulting in the restoration of T and NK cell functions [67]. Nakahara et al. [68] suggested that the effect of cyclophosphamide on T-regs relied on its capacity to eliminate CD8^+^ resident DC, which induce T-reg activation. Another report suggested that T-regs present low levels of ATP, which attenuated the synthesis of glutathione, leading to a decrease of cyclophosphamide detoxification, thus increasing the sensitivity of T-reg cells to low-dose cyclophosphamide [69].

Gemcitabine reduced T-regs in patients with pancreatic cancer [47]. Furthermore, the percentage of T-regs was significantly reduced following gemcitabine plus cisplatin chemotherapy in a small series of 40 patients with advanced NSCLC [70].

For chemotherapies used for digestive cancer, oxaliplatin increases T-cell infiltration in tumors, including T-regs [71], while 5-FU has no apparent effect on T-regs [72]. Combotherapy with 5FU plus oxaliplatin (FOLFOX) enhances antitumor immunity via suppression of T-regs [73].

Low-dose metronomic regimens of temozolomide, an alkylating agent, can reduce T-regs and impair T-reg suppressive activity in glioma-bearing rats [74]. Temozolomide was also show to reduce circulating T-reg in advanced melanoma patients [45].

In a mouse model, paclitaxel has been shown to decrease T-reg levels, as well as reduce their inhibitory function [75]. In a clinical study of 40 NSCLC patients, docetaxel-based chemotherapy significantly decreased T-regs in 17 patients (17/40) [76]. Another study showed that microtubule inhibitors, including taxanes and vinca alkaloids, increased the relative abundance of circulating T cells, with a significant increase in the ratio of effector T-cells to T-regs [77]. The selective induction of T-reg apoptosis by taxanes was attributed to the upregulation of the cell death receptor FAS in T-regs, but paclitaxel did not decrease the size of other subsets including effector cells [78].

### 3.3. Depletion of TAM

Tumor-associated macrophages (TAM) play a major role at the tumor interface and regulate local immune response. TAM are classically subdivided into two types: the antitumoral TAM-1 and pro-tumoral TAM-2. Despite the important prognostic role of TAM in many cancers [79], few chemotherapies are known to deplete or affect TAM function.

Trabectedin—approved by the European Medicines Agency for soft tissue sarcoma and ovarian cancer—is a DNA binder that causes DNA damage, but also has immunological effects. Reduced TAM infiltration was observed in biopsies from sarcoma patients treated with trabectedin [80]. Recently, we showed that TAS 102, an oral antimetabolite used in metastatic colon cancer, decreases TAM, especially TAM-2 [36].

Conversely, some chemotherapy regimens have demonstrated an ability to promote TAM activity. Platinum-based therapy enhances M2 polarization. paclitaxel increases CSF1 and CCL2 production [81] leading to recruitment of macrophages and polarization towards an immunosuppressive phenotype [82].

## 4. Combination of Chemotherapy and Immunotherapy

Currently, there are only a few approved combinations of chemotherapy plus immunotherapy: cisplatin-pemetrexed, or carboplatin–paclitaxel plus pembrolizumab as a first line therapy for advanced/metastatic NSCLC [5], atezolizumab, carboplatin and etoposide for small-cell lung cancer [83] and nab-paclitaxel or paclitaxel with atezolizumab as a first line therapy for advanced/metastatic breast cancer [84]. Adjuvant treatment with durvalumab is also recommended for stage III NSCLC after radio-chemotherapy [85]. Many Phase III trials are ongoing in various diseases and will change the face of oncology in multiple indications.

Based on this review, we purport that choice of the optimal chemotherapy agent (or regimen) to be combined with immunotherapy in the future should take account of the effect on ICD and on immunosuppressive cells. In digestive cancer, the FOLFOX regimen looks very promising because of its capacity to lead to ICD by oxaliplatin and depletion of MDSC by 5-FU [54]. Preliminary results of the MEDETREME trial (FOLFOX + durvalumab + tremelimumab) have shown promising results in metastatic microsatellite-stable colorectal cancer [55]. In FOLFIRI, the effect of irinotecan on the immune system is less well known. Interestingly, using TAS-102 to deplete TAM in combination with oxaliplatin to induce ICD may represent a combination worthy of further investigation and development.

In gynecologic cancers, doublets with carboplatin are currently used. TIGIT induction on T cells in preclinical models with carboplatin resistance [66] provides a strong rationale to combine this carboplatin with anti-TIGIT therapy. While carboplatin is used in first line in all gynecologic cancers, the choice of the doublet of chemotherapy will be important for future combinations with immune-checkpoint inhibitors. Paclitaxel, gemcitabine and liposomal doxorubicin could be used and could have differential effects on the immune system. Paclitaxel depletes MDSC and T-regs, gemcitabine depletes MDSC and modifies the polarization of TAM, while liposomal doxorubicin is a strong ICD inducer. Furthermore, previous results using trabectedin in sarcoma have shown its ability to deplete TAM [80]. This drug could also be used in ovarian cancer and may be of interest thanks to its capacity to modulate the immunosuppressive microenvironment Based on preclinical data, carboplatin and liposomal doxorubicin seems to be the most promising option for use in combination with immune checkpoint inhibitors. Poly ADP ribose polymerase (PARP) inhibitors represent another interesting option, but are beyond the scope of this review, which focuses on cytotoxic drugs.

Based on in vitro data, pemetrexed appears to be the best chemotherapy to associate with platinum in lung cancer [37,38]. In vivo analyses are needed to confirm this hypothesis and validate this choice as first line therapy in combination with pembrolizumab.

In breast cancer, the ASCO and ESMO guidelines recommend using monotherapy of a cytotoxic agent in metastatic disease. The choice of chemotherapy to be associated with immune checkpoint inhibitors will be important in this context also. Currently, paclitaxel and nab-paclitaxel are preferentially chosen, because Phase III clinical trials were developed with these regimens. Doxorubicin used as monotherapy or in combination with platinum therapy will be of interest for future clinical trials. Preliminary data in a small clinical trial [25] showed that the best response rate and the most potent induction of immune response were observed with a combination of anti PD-1 with a platin or doxorubicin.

In urological cancer, a combination of gemcitabine and platinum or anthracycline with platinum (like in the MVAC regimen) may be of interest for urothelial cancer, while targeted therapies are probably the best partner to be combined with immune checkpoint inhibitors for renal carcinoma [86].

## 5. Conclusions

Although some cancers are known to be sensitive to immune checkpoint inhibitors (such as lung cancer, melanoma, microsatellite instability tumors), others have shown disappointing results (ovarian, microsatellite stable colonic cancer, pancreatic carcinoma). Several biomarkers have been developed to understand resistance mechanism to immunotherapy. In most contexts, it seems that preexisting immune response is a prerequisite to observe any efficacy of immune checkpoint inhibitors. Accordingly, tumors are defined as “hot tumors” with high levels of immune infiltrate, versus “cold tumors”, with only hot tumors known to yield benefit from monotherapy with checkpoint inhibitors.

Cytotoxic chemotherapy agents have classically been administered to fight against cancer. Although most drugs have detrimental effects on immune homeostasis (lymphopenia), they can be useful to increase antitumor immunogenicity, thus sensitizing cancer cells to immunotherapy in some contexts. Target therapies [87] and radiation therapy [88] were described to be efficient on ICD induction and could used in the same way. These data provide a rationale for combining chemotherapy with immune checkpoint inhibitors, in order to modulate and prepare the tumor microenvironment. Broadening our knowledge of the immune effects of chemotherapies is necessary, both in preclinical models and in patients. Induction of ICD is a good way to promote and enhance a CD8-dependent immune response in poorly immunogenic tumor; elimination of immunosuppressive cells could also be used to favor CD8 immune response. The strategy that consists of testing the effect of a classical combination of chemotherapies on ICD and immunosuppressive cells used in the clinical setting is an unmet need to advance the development of chemoimmunotherapy.

## Figures and Tables

**Table 1:** ICD and depletion of immunosuppressive cells by chemotherapies.

Drug	ICD Associated DAMPs				Action on Immunosuppressive Cells			References
	CRT Exposure	ATP Release	HMGB1 Release	Type 1 IFN Secretion	MDSC	Treg	TAM	
Cisplatin	−	+	+	unknown	depletion			[23,24]
Carboplatin	+/−	+	+/−	unknown	activation			[10,25,26]
Oxaliplatin	+	+	?	unknown	depletion			[27,28]
Gemcitabine	−	+/−	+	unknown	depletion		polarization	[29,30,31]
5FU	+	−	+	unknown	depletion			[32,33,34]
TAS 102	+	+	+	+			depletion	[35]
Pemetrexed	+	+	+	+				[36,37]
Anthracyclines	+	+	+	+	depletion			[38,39,40]
Cyclophosphamide	+	+	+	+		depletion		[15,20]
Bleomycin	+	+	+	+		increase		[41]
Irinotecan	unknown	unknown	+	unknown				[42]
Docetaxel	+	−	+	unknown	differentiation			[43,44,45]
Paclitaxel	+	+	+	unknown	depletion			[46,47]

## References

[1] Borghaei H., Paz-Ares L., Horn L., Spigel D.R., Steins M., Ready N.E., Chow L.Q., Vokes E.E., Felip E., Holgado E. (2015). Nivolumab versus Docetaxel in Advanced Nonsquamous Non–Small-Cell Lung Cancer. N. Engl. J. Med..

[2] Fumet J.-D., Richard C., Ledys F., Klopfenstein Q., Joubert P., Routy B., Truntzer C., Gagné A., Hamel M.-A., Guimaraes C.F. (2018). Prognostic and predictive role of CD8 and PD-L1 determination in lung tumor tissue of patients under anti-PD-1 therapy. Br. J. Cancer.

[3] Cristescu R., Mogg R., Ayers M., Albright A., Murphy E., Yearley J., Sher X., Liu X.Q., Lu H., Nebozhyn M. (2018). Pan-tumor genomic biomarkers for PD-1 checkpoint blockade–based immunotherapy. Science.

[4] Fares C.M., Van Allen E.M., Drake C.G., Allison J.P., Hu-Lieskovan S. (2019). Mechanisms of Resistance to Immune Checkpoint Blockade: Why Does Checkpoint Inhibitor Immunotherapy Not Work for All Patients?. Am. Soc. Clin. Oncol. Educ. Book.

[5] Gandhi L., Rodríguez-Abreu D., Gadgeel S., Esteban E., Felip E., De Angelis F., Domine M., Clingan P., Hochmair M.J., Powell S.F. (2018). Pembrolizumab plus Chemotherapy in Metastatic Non–Small-Cell Lung Cancer. N. Engl. J. Med..

[6] Galluzzi L., Vitale I., Aaronson S.A., Abrams J.M., Adam D., Agostinis P., Alnemri E.S., Altucci L., Amelio I., Andrews D.W. (2018). Molecular mechanisms of cell death: Recommendations of the Nomenclature Committee on Cell Death 2018. Cell Death Differ..

[7] Duan Q., Zhang H., Zheng J., Zhang L. (2020). Turning Cold into Hot: Firing up the Tumor Microenvironment. Trends Cancer.

[8] Galluzzi L., Buqué A., Kepp O., Zitvogel L., Kroemer G. (2017). Immunogenic cell death in cancer and infectious disease. Nat. Rev. Immunol..

[9] Vanpouille-Box C., Alard A., Aryankalayil M.J., Sarfraz Y., Diamond J.M., Schneider R.J., Inghirami G., Coleman C.N., Formenti S.C., Demaria S. (2017). DNA exonuclease Trex1 regulates radiotherapy-induced tumour immunogenicity. Nat. Commun..

[10] Minute L., Teijeira A., Sanchez-Paulete A.R., Ochoa M.C., Alvarez M., Otano I., Etxeberrria I., Bolaños E., Azpilikueta A., Garasa S. (2020). Cellular cytotoxicity is a form of immunogenic cell death. J. Immunother. Cancer.

[11] Obeid M., Tesniere A., Ghiringhelli F., Fimia G.M., Apetoh L., Perfettini J.-L., Castedo M., Mignot G., Panaretakis T., Casares N. (2007). Calreticulin exposure dictates the immunogenicity of cancer cell death. Nat. Med..

[12] Gardai S.J., McPhillips K.A., Frasch S.C., Janssen W.J., Starefeldt A., Murphy-Ullrich J.E., Bratton D.L., Oldenborg P.-A., Michalak M., Henson P.M. (2005). Cell-Surface Calreticulin Initiates Clearance of Viable or Apoptotic Cells through trans-Activation of LRP on the Phagocyte. Cell.

[13] Michaud M., Martins I., Sukkurwala A.Q., Adjemian S., Ma Y., Pellegatti P., Shen S., Kepp O., Scoazec M., Mignot G. (2011). Autophagy-Dependent Anticancer Immune Responses Induced by Chemotherapeutic Agents in Mice. Science.

[14] Scaffidi P., Misteli T., Bianchi M.E. (2002). Release of chromatin protein HMGB1 by necrotic cells triggers inflammation. Nature.

[15] Conte A., Paladino S., Bianco G., Fasano D., Gerlini R., Tornincasa M., Renna M., Fusco A., Tramontano D., Pierantoni G.M. (2017). High mobility group A1 protein modulates autophagy in cancer cells. Cell Death Differ..

[16] Sistigu A., Yamazaki T., Vacchelli E., Chaba K., Enot D.P., Adam J., Vitale I., Goubar A., Baracco E.E., Remédios C. (2014). Cancer cell–autonomous contribution of type I interferon signaling to the efficacy of chemotherapy. Nat. Med..

[17] Vacchelli E., Ma Y., Baracco E.E., Sistigu A., Enot D.P., Pietrocola F., Yang H., Adjemian S., Chaba K., Semeraro M. (2015). Chemotherapy-induced antitumor immunity requires formyl peptide receptor 1. Science.

[18] Elliott M.R., Chekeni F.B., Trampont P.C., Lazarowski E.R., Kadl A., Walk S.F., Park D., Woodson R.I., Ostankovich M., Sharma P. (2009). Nucleotides released by apoptotic cells act as a find-me signal to promote phagocytic clearance. Nature.

[19] Ghiringhelli F., Apetoh L., Tesniere A., Aymeric L., Ma Y., Ortiz C., Vermaelen K., Panaretakis T., Mignot G., Ullrich E. (2009). Activation of the NLRP3 inflammasome in dendritic cells induces IL-1β–dependent adaptive immunity against tumors. Nat. Med..

[20] Yanai H., Ban T., Wang Z., Choi M.K., Kawamura T., Negishi H., Nakasato M., Lu Y., Hangai S., Koshiba R. (2009). HMGB proteins function as universal sentinels for nucleic-acid-mediated innate immune responses. Nature.

[21] Huang J., Xie Y., Sun X., Zeh H.J., Kang R., Lotze M.T., Tang D. (2015). DAMPs, Ageing, and Cancer: The ‘DAMP Hypothesis’. Ageing Res. Rev..

[22] Krysko O., Løve Aaes T., Bachert C., Vandenabeele P., Krysko D.V. (2013). Many faces of DAMPs in cancer therapy. Cell Death Dis..

[23] Hato S.V., Khong A., de Vries I.J.M., Lesterhuis W.J. (2014). Molecular Pathways: The Immunogenic Effects of Platinum-Based Chemotherapeutics. Clin. Cancer Res..

[24] Sevko A., Umansky V. (2012). Myeloid-Derived Suppressor Cells Interact with Tumors in Terms of Myelopoiesis, Tumorigenesis and Immunosuppression: Thick as Thieves. J. Cancer.

[25] Voorwerk L., Slagter M., Horlings H.M., Sikorska K., van de Vijver K.K., de Maaker M., Nederlof I., Kluin R.J.C., Warren S., Ong S. (2019). Immune induction strategies in metastatic triple-negative breast cancer to enhance the sensitivity to PD-1 blockade: The TONIC trial. Nat. Med..

[26] Huang X., Cui S., Shu Y. (2016). Cisplatin selectively downregulated the frequency and immunoinhibitory function of myeloid-derived suppressor cells in a murine B16 melanoma model. Immunol. Res..

[27] Golden E.B., Frances D., Pellicciotta I., Demaria S., Helen Barcellos-Hoff M., Formenti S.C. (2014). Radiation fosters dose-dependent and chemotherapy-induced immunogenic cell death. Oncoimmunology.

[28] Alizadeh D., Trad M., Hanke N.T., Larmonier C.B., Janikashvili N., Bonnotte B., Katsanis E., Larmonier N. (2014). Doxorubicin Eliminates Myeloid-Derived Suppressor Cells and Enhances the Efficacy of Adoptive T-Cell Transfer in Breast Cancer. Cancer Res..

[29] Liu W.M., Fowler D.W., Smith P., Dalgleish A.G. (2010). Pre-treatment with chemotherapy can enhance the antigenicity and immunogenicity of tumours by promoting adaptive immune responses. Br. J. Cancer.

[30] Annels N.E., Shaw V.E., Gabitass R.F., Billingham L., Corrie P., Eatock M., Valle J., Smith D., Wadsley J., Cunningham D. (2014). The effects of gemcitabine and capecitabine combination chemotherapy and of low-dose adjuvant GM-CSF on the levels of myeloid-derived suppressor cells in patients with advanced pancreatic cancer. Cancer Immunol. Immunother..

[31] Kanterman J., Sade-Feldman M., Biton M., Ish-Shalom E., Lasry A., Goldshtein A., Hubert A., Baniyash M. (2014). Adverse Immunoregulatory Effects of 5FU and CPT11 Chemotherapy on Myeloid-Derived Suppressor Cells and Colorectal Cancer Outcomes. Cancer Res..

[32] Yin H., Yang X., Gu W., Liu Y., Li X., Huang X., Zhu X., Tao Y., Gou X., He W. (2017). HMGB1-mediated autophagy attenuates gemcitabine-induced apoptosis in bladder cancer cells involving JNK and ERK activation. Oncotarget.

[33] Yamamura Y., Tsuchikawa T., Miyauchi K., Takeuchi S., Wada M., Kuwatani T., Kyogoku N., Kuroda A., Maki T., Shichinohe T. (2015). The key role of calreticulin in immunomodulation induced by chemotherapeutic agents. Int. J. Clin. Oncol..

[34] Hodge J.W., Garnett C.T., Farsaci B., Palena C., Tsang K.-Y., Ferrone S., Gameiro S.R. (2013). Chemotherapy-induced immunogenic modulation of tumor cells enhances killing by cytotoxic T lymphocytes and is distinct from immunogenic cell death. Int. J. Cancer.

[35] Cottone L., Capobianco A., Gualteroni C., Perrotta C., Bianchi M.E., Rovere-Querini P., Manfredi A.A. (2015). 5-Fluorouracil causes leukocytes attraction in the peritoneal cavity by activating autophagy and HMGB1 release in colon carcinoma cells. Int. J. Cancer.

[36] Limagne E., Thibaudin M., Nuttin L., Spill A., Dérangère V., Fumet J.-D., Amellal N., Peranzoni E., Cattan V., Ghiringhelli F. (2019). Trifluridine/tipiracil plus oxaliplatin improves PD-1 blockade in colorectal cancer by inducing immunogenic cell death and depleting macrophages. Cancer Immunol. Res..

[37] Novosiadly R., Schaer D., Amaladas N., Rasmussen E., Lu Z.H., Sonyi A., Carpenito C., Li Y., Luo S., Capen A. (2018). Abstract 4549: Pemetrexed enhances anti-tumor efficacy of PD1 pathway blockade by promoting intra tumor immune response via immunogenic tumor cell death and T cell intrinsic mechanisms. Cancer Res..

[38] Schaer D.A., Geeganage S., Amaladas N., Lu Z.H., Rasmussen E.R., Sonyi A., Chin D., Capen A., Li Y., Meyer C.M. (2019). The folate pathway inhibitor pemetrexed pleiotropically enhances effects of cancer immunotherapy. Clin. Cancer Res..

[39] Casares N., Pequignot M.O., Tesniere A., Ghiringhelli F., Roux S., Chaput N., Schmitt E., Hamai A., Hervas-Stubbs S., Obeid M. (2005). Caspase-dependent immunogenicity of doxorubicin-induced tumor cell death. J. Exp. Med..

[40] Liechtenstein T., Perez-Janices N., Gato M., Caliendo F., Kochan G., Blanco-Luquin I., Van der Jeught K., Arce F., Guerrero-Setas D., Fernandez-Irigoyen J. (2014). A highly efficient tumor-infiltrating MDSC differentiation system for discovery of anti-neoplastic targets, which circumvents the need for tumor establishment in mice. Oncotarget.

[41] Giglio P., Gagliardi M., Tumino N., Antunes F., Smaili S., Cotella D., Santoro C., Bernardini R., Mattei M., Piacentini M. (2018). PKR and GCN2 stress kinases promote an ER stress-independent eIF2α phosphorylation responsible for calreticulin exposure in melanoma cells. OncoImmunology.

[42] Bugaut H., Bruchard M., Berger H., Derangère V., Odoul L., Euvrard R., Ladoire S., Chalmin F., Végran F., Rébé C. (2013). Bleomycin Exerts Ambivalent Antitumor Immune Effect by Triggering Both Immunogenic Cell Death and Proliferation of Regulatory T Cells. PLoS ONE.

[43] Lau T.-S., Chan L.K.-Y., Man G.C.-W., Kwong J. (2019). Abstract 1232: Paclitaxel induces immunogenic cell death in ovarian cancer via TLR4-independent and dependent pathways. Cancer Res..

[44] Le H.K., Graham L., Cha E., Morales J.K., Manjili M.H., Bear H.D. (2009). Gemcitabine directly inhibits myeloid derived suppressor cells in BALB/c mice bearing 4T1 mammary carcinoma and augments expansion of T cells from tumor-bearing mice. Int. Immunopharmacol..

[45] Ridolfi L., Petrini M., Granato A.M., Gentilcore G., Simeone E., Ascierto P.A., Pancisi E., Ancarani V., Fiammenghi L., Guidoboni M. (2013). Low-dose temozolomide before dendritic-cell vaccination reduces (specifically) CD4+CD25++Foxp3+ regulatory T-cells in advanced melanoma patients. J. Transl. Med..

[46] Pfannenstiel L.W., Lam S.S.K., Emens L.A., Jaffee E.M., Armstrong T.D. (2010). Paclitaxel enhances early dendritic cell maturation and function through TLR4 signaling in mice. Cell Immunol..

[47] Eriksson E., Wenthe J., Irenaeus S., Loskog A., Ullenhag G. (2016). Gemcitabine reduces MDSCs, tregs and TGFβ-1 while restoring the teff/treg ratio in patients with pancreatic cancer. J. Transl. Med..

[48] Schiavoni G., Sistigu A., Valentini M., Mattei F., Sestili P., Spadaro F., Sanchez M., Lorenzi S., D’Urso M.T., Belardelli F. (2011). Cyclophosphamide Synergizes with Type I Interferons through Systemic Dendritic Cell Reactivation and Induction of Immunogenic Tumor Apoptosis. Cancer Res..

[49] Sistigu A., Viaud S., Chaput N., Bracci L., Proietti E., Zitvogel L. (2011). Immunomodulatory effects of cyclophosphamide and implementations for vaccine design. Semin. Immunopathol..

[50] Martins I., Kepp O., Schlemmer F., Adjemian S., Tailler M., Shen S., Michaud M., Menger L., Gdoura A., Tajeddine N. (2011). Restoration of the immunogenicity of cisplatin-induced cancer cell death by endoplasmic reticulum stress. Oncogene.

[51] Pfirschke C., Engblom C., Rickelt S., Cortez-Retamozo V., Garris C., Pucci F., Yamazaki T., Colame V.P., Newton A., Redouane Y. (2016). Immunogenic chemotherapy sensitizes tumors to checkpoint blockade therapy. Immunity.

[52] Aranda F., Bloy N., Pesquet J., Petit B., Chaba K., Sauvat A., Kepp O., Khadra N., Enot D., Pfirschke C. (2015). Immune-dependent antineoplastic effects of cisplatin plus pyridoxine in non-small-cell lung cancer. Oncogene.

[53] Tesniere A., Schlemmer F., Boige V., Kepp O., Martins I., Ghiringhelli F., Aymeric L., Michaud M., Apetoh L., Barault L. (2010). Immunogenic death of colon cancer cells treated with oxaliplatin. Oncogene.

[54] Dosset M., Vargas T.R., Lagrange A., Boidot R., Végran F., Roussey A., Chalmin F., Dondaine L., Paul C., Marie-Joseph E.L. (2018). PD-1/PD-L1 pathway: An adaptive immune resistance mechanism to immunogenic chemotherapy in colorectal cancer. OncoImmunology.

[55] Ghiringhelli F., Chibaudel B., Taieb J., Bennouna J., Martin-Babau J., Fonck M., Borg C., Cohen R., Thibaudin M., Limagne E. (2020). Durvalumab and tremelimumab in combination with FOLFOX in patients with RAS-mutated, microsatellite-stable, previously untreated metastatic colorectal cancer (MCRC): Results of the first intermediate analysis of the phase Ib/II MEDETREME trial. JCO.

[56] Geary S.M., Lemke C.D., Lubaroff D.M., Salem A.K. (2013). The Combination of a Low-Dose Chemotherapeutic Agent, 5-Fluorouracil, and an Adenoviral Tumor Vaccine Has a Synergistic Benefit on Survival in a Tumor Model System. PLoS ONE.

[57] Keyvani-Ghamsari S., Rabbani-Chadegani A., Sargolzaei J., Shahhoseini M. (2017). Effect of irinotecan on HMGB1, MMP9 expression, cell cycle, and cell growth in breast cancer (MCF-7) cells. Tumor Biol..

[58] Wan S., Pestka S., Jubin R.G., Lyu Y.L., Tsai Y.-C., Liu L.F. (2012). Chemotherapeutics and Radiation Stimulate MHC Class I Expression through Elevated Interferon-beta Signaling in Breast Cancer Cells. PLoS ONE.

[59] Volk-Draper L., Hall K., Griggs C., Rajput S., Kohio P., DeNardo D., Ran S. (2014). Paclitaxel Therapy Promotes Breast Cancer Metastasis in a TLR4-Dependent Manner. Cancer Res..

[60] Petty A.J., Yang Y. (2017). Tumor-associated macrophages: Implications in cancer immunotherapy. Immunotherapy.

[61] Vincent J., Mignot G., Chalmin F., Ladoire S., Bruchard M., Chevriaux A., Martin F., Apetoh L., Rébé C., Ghiringhelli F. (2010). 5-Fluorouracil Selectively Kills Tumor-Associated Myeloid-Derived Suppressor Cells Resulting in Enhanced T Cell–Dependent Antitumor Immunity. Cancer Res..

[62] Bruchard M., Ghiringhelli F. (2014). Modulation de l’immunosuppression par des chimiothérapies et identification de nouvelles cibles thérapeutiques. Bull. Cancer.

[63] Limagne E., Euvrard R., Thibaudin M., Rébé C., Derangère V., Chevriaux A., Boidot R., Végran F., Bonnefoy N., Vincent J. (2016). Accumulation of MDSC and Th17 Cells in Patients with Metastatic Colorectal Cancer Predicts the Efficacy of a FOLFOX–Bevacizumab Drug Treatment Regimen. Cancer Res..

[64] Kodumudi K.N., Woan K., Gilvary D.L., Sahakian E., Wei S., Djeu J.Y. (2010). A Novel Chemoimmunomodulating Property of Docetaxel: Suppression of Myeloid-Derived Suppressor Cells in Tumor Bearers. Clin. Cancer Res..

[65] Kim N., Kim Y. (2018). Oxaliplatin regulates myeloid-derived suppressor cell-mediated immunosuppression via downregulation of nuclear factor-κB signaling. Cancer Med..

[66] Anestakis D., Petanidis S., Domvri K., Tsavlis D., Zarogoulidis P., Katopodi T. (2020). Carboplatin chemoresistance is associated with CD11b+/Ly6C+ myeloid release and upregulation of TIGIT and LAG3/CD160 exhausted T cells. Mol. Immunol..

[67] Ghiringhelli F., Menard C., Puig P.E., Ladoire S., Roux S., Martin F., Solary E., Le Cesne A., Zitvogel L., Chauffert B. (2007). Metronomic cyclophosphamide regimen selectively depletes CD4+CD25+ regulatory T cells and restores T and NK effector functions in end stage cancer patients. Cancer Immunol. Immunother..

[68] Nakahara T., Uchi H., Lesokhin A.M., Avogadri F., Rizzuto G.A., Hirschhorn-Cymerman D., Panageas K.S., Merghoub T., Wolchok J.D., Houghton A.N. (2010). Cyclophosphamide enhances immunity by modulating the balance of dendritic cell subsets in lymphoid organs. Blood.

[69] Zhao J., Cao Y., Lei Z., Yang Z., Zhang B., Huang B. (2010). Selective Depletion of CD4+CD25+Foxp3+ Regulatory T Cells by Low-Dose Cyclophosphamide Is Explained by Reduced Intracellular ATP Levels. Cancer Res..

[70] Chen C., Chen Z., Chen D., Zhang B., Wang Z., Le H. (2015). Suppressive effects of gemcitabine plus cisplatin chemotherapy on regulatory T cells in nonsmall-cell lung cancer. J. Int. Med. Res..

[71] Stojanovska V., Prakash M., McQuade R., Fraser S., Apostolopoulos V., Sakkal S., Nurgali K. Oxaliplatin Treatment Alters Systemic Immune Responses. https://www.hindawi.com/journals/bmri/2019/4650695/.

[72] Wang J., Peng L., Zhang R., Zheng Z., Chen C., Cheung K.L., Cui M., Bian G., Xu F., Chiang D. (2016). 5-Fluorouracil targets thymidylate synthase in the selective suppression of T H 17 cell differentiation. Oncotarget.

[73] Maeda K., Hazama S., Tokuno K., Kan S., Maeda Y., Watanabe Y., Kamei R., Shindo Y., Maeda N., Yoshimura K. (2011). Impact of Chemotherapy for Colorectal Cancer on Regulatory T-Cells and Tumor Immunity. Anticancer Res..

[74] Banissi C., Ghiringhelli F., Chen L., Carpentier A.F. (2009). Treg depletion with a low-dose metronomic temozolomide regimen in a rat glioma model. Cancer Immunol. Immunother..

[75] Vicari A.P., Luu R., Zhang N., Patel S., Makinen S.R., Hanson D.C., Weeratna R.D., Krieg A.M. (2009). Paclitaxel reduces regulatory T cell numbers and inhibitory function and enhances the anti-tumor effects of the TLR9 agonist PF-3512676 in the mouse. Cancer Immunol. Immunother..

[76] Li J.-Y., Duan X.-F., Wang L.-P., Xu Y.-J., Huang L., Zhang T.-F., Liu J.-Y., Li F., Zhang Z., Yue D.-L. Selective Depletion of Regulatory T Cell Subsets by Docetaxel Treatment in Patients with Nonsmall Cell Lung Cancer. https://www.hindawi.com/journals/jir/2014/286170/.

[77] Roselli M., Cereda V., di Bari M.G., Formica V., Spila A., Jochems C., Farsaci B., Donahue R., Gulley J.L., Schlom J. (2013). Effects of conventional therapeutic interventions on the number and function of regulatory T cells. Oncoimmunology.

[78] Zhang L., Dermawan K., Jin M., Liu R., Zheng H., Xu L., Zhang Y., Cai Y., Chu Y., Xiong S. (2008). Differential impairment of regulatory T cells rather than effector T cells by paclitaxel-based chemotherapy. Clin. Immunol..

[79] Mantovani A., Marchesi F., Malesci A., Laghi L., Allavena P. (2017). Tumor-Associated Macrophages as Treatment Targets in Oncology. Nat. Rev. Clin. Oncol..

[80] Germano G., Frapolli R., Belgiovine C., Anselmo A., Pesce S., Liguori M., Erba E., Uboldi S., Zucchetti M., Pasqualini F. (2013). Role of Macrophage Targeting in the Antitumor Activity of Trabectedin. Cancer Cell.

[81] Mantovani A., Allavena P. (2015). The interaction of anticancer therapies with tumor-associated macrophages. J. Exp. Med..

[82] DeNardo D.G., Brennan D.J., Rexhepaj E., Ruffell B., Shiao S.L., Madden S.F., Gallagher W.M., Wadhwani N., Keil S.D., Junaid S.A. (2011). Leukocyte Complexity Predicts Breast Cancer Survival and Functionally Regulates Response to Chemotherapy. Cancer Discov..

[83] Horn L., Mansfield A.S., Szczęsna A., Havel L., Krzakowski M., Hochmair M.J., Huemer F., Losonczy G., Johnson M.L., Nishio M. (2018). First-Line Atezolizumab plus Chemotherapy in Extensive-Stage Small-Cell Lung Cancer. N. Engl. J. Med..

[84] Schmid P., Adams S., Rugo H.S., Schneeweiss A., Barrios C.H., Iwata H., Diéras V., Hegg R., Im S.-A., Shaw Wright G. (2018). Atezolizumab and Nab-Paclitaxel in Advanced Triple-Negative Breast Cancer. N. Engl. J. Med..

[85] Antonia S.J., Villegas A., Daniel D., Vicente D., Murakami S., Hui R., Yokoi T., Chiappori A., Lee K.H., de Wit M. (2017). Durvalumab after Chemoradiotherapy in Stage III Non–Small-Cell Lung Cancer. N. Engl. J. Med..

[86] Rini B.I., Plimack E.R., Stus V., Gafanov R., Hawkins R., Nosov D., Pouliot F., Alekseev B., Soulières D., Melichar B. (2019). Pembrolizumab plus Axitinib versus Sunitinib for Advanced Renal-Cell Carcinoma. N. Engl. J. Med..

[87] Vanneman M., Dranoff G. (2012). Combining Immunotherapy and Targeted Therapies in Cancer Treatment. Nat. Rev. Cancer.

[88] Ko E.C., Formenti S.C. (2018). Radiotherapy and checkpoint inhibitors: A winning new combination?. Ther. Adv. Med. Oncol..

